# The Role of Sulforaphane in Epigenetic Mechanisms, Including Interdependence between Histone Modification and DNA Methylation

**DOI:** 10.3390/ijms161226195

**Published:** 2015-12-12

**Authors:** Agnieszka Kaufman-Szymczyk, Grzegorz Majewski, Katarzyna Lubecka-Pietruszewska, Krystyna Fabianowska-Majewska

**Affiliations:** 1 Department of Biomedical Chemistry, Faculty of Health Sciences, Medical University of Lodz, 6/8 Mazowiecka St., 92-215 Lodz, Poland; katarzyna.lubecka-pietruszewska@umed.lodz.pl (K.L.-P.); krystyna.fabianowska-majewska@umed.lodz.pl (K.F.-M.); 2 Faculty of Public Health, University of Social Sciences in Lodz, 9 Sienkiewicza St., 90-113 Lodz, Poland; kitmaj@toya.net.pl

**Keywords:** cruciferous vegetables, isothiocyanates, sulforaphane, epigenetic cancer chemoprevention

## Abstract

Carcinogenesis as well as cancer progression result from genetic and epigenetic changes of the genome that leads to dysregulation of transcriptional activity of genes. Epigenetic mechanisms in cancer cells comprise (i) post-translation histone modification (*i.e.*, deacetylation and methylation); (ii) DNA global hypomethylation; (iii) promoter hypermethylation of tumour suppressor genes and genes important for cell cycle regulation, cell differentiation and apoptosis; and (iv) posttranscriptional regulation of gene expression by noncoding microRNA. These epigenetic aberrations can be readily reversible and responsive to both synthetic agents and natural components of diet. A source of one of such diet components are cruciferous vegetables, which contain high levels of a number of glucosinolates and deliver, after enzymatic hydrolysis, sulforaphane and other bioactive isothiocyanates, that are involved in effective up-regulation of transcriptional activity of certain genes and also in restoration of active chromatin structure. Thus a consumption of cruciferous vegetables, treated as a source of isothiocyanates, seems to be potentially useful as an effective cancer preventive factor or as a source of nutrients improving efficacy of standard chemotherapies. In this review an attempt is made to elucidate the role of sulforaphane in regulation of gene promoter activity through a direct down-regulation of histone deacetylase activity and alteration of gene promoter methylation in indirect ways, but the sulforaphane influence on non-coding micro-RNA will not be a subject of this review.

## 1. Introduction

On account of high morbidity and mortality, cancer prevention has become a public priority in almost every country of the world. In an early step of carcinogenesis, as well as in progression of cancers, not only genetic changes but also epigenetic dysregulation processes are observed. Epigenetic mechanisms comprise (i) post-translation histone modifications (*i.e.*, histone acetylation/deacetylation and methylation); (ii) DNA global hypomethylation; (iii) promoter hypermethylation of tumour suppressor genes and genes important mainly for regulation of cell cycle; and (iv) posttranscriptional regulation of gene expression by noncoding microRNAs. Epigenetic aberrations can be relatively readily reversible and responsive to environmental factors, including diet, they have been identified as auspicious targets for new, additional strategies of carcinogenesis prevention or for improvement of efficacy of current standard chemotherapy. Many natural dietary agents, consisting of bioactive compounds, have been shown to be effective nutraceuticals in cancer prevention due to mediation between favorable epigenetic changes [1]. Epidemiological study results indicate that dietary consumption of cruciferous vegetables—such as broccoli, broccoli sprouts, cabbage or kale—may reduce the risk of many common cancers, including prostate, breast, lung and colorectal cancers [2]. These cruciferous vegetables contain high levels of different glucosinolates, which, after enzymatic hydrolysis by myrosinase (β-thioglucosidase—an enzyme present in plants or in intestinal microbes), deliver—in neutral pH—bioactive isothiocyanates e.g., sulforaphane (SFN, 1-siothiocyanato-4-methylsulfinylbutane) (Table 1). During the last decade it has been documented several times that the chemopreventive properties of isothiocyanates and also their metabolites (some products of the mercapturic acid pathway) are involved in multiple mechanisms whose consequences are: growth inhibition of various types of cancer cells, e.g., MCF-7 breast cancer cells [3], inhibition of proliferation of several melanoma cell lines [4], and induction of apoptosis of some cancer cell lines, e.g., MG-63 osteosarcoma and breast cancer cells [5,6]. Moreover, SFN can inhibit migration and invasion of glioblastoma cells [7]. The plant compound has also been taken into consideration for treatment of hematological malignancies [8,9]. Additionally, SFN can protect cells against toxic effects of carcinogens by inhibition of activity of enzymes involved in carcinogen activation as well as by inducing activities of several cytoprotective phase 2 enzymes, e.g., glutathione S-transferases (GSTs), UDP-glucuronosyl transferases (UGTs), NAD(P)H: quinone oxidoreductase 1 (NQO1) *etc.*, which protects cells from DNA damage, promotes the removal of carcinogens and generally leads to detoxification [10].

**Table 1:** Chemical structure of selected isothiocyanates and content of their glucosinolate precursors in raw cruciferous vegetables.

Isothiocyanate	Chemical Structure	Glucosinolate—Isothiocyanate Precursor	Food Sources	Total Concentration (mg/100 g)
Sulforaphane	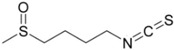	Glucoraphanin	Broccoli	61
			Brussels sprouts	236
			Cabbage	78
Allyl isothiocyanate (AITC)	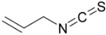	Sinigrin	Broccoli	61
			Brussels sprouts	236
			Cabbage	78
			Mustard greens	282
Benzyl isothiocyanate (BITC)	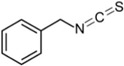	Glucotropaeolin	Cabbage	78
			Garden cress	392
Phenethyl isothiocyanate (PEITC)	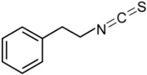	Gluconasturtiin	Watercress	94

For the benefit of consumers, dieticians and clinical oncologists this review summarizes the hitherto reported findings of *in vitro* and *in vivo* studies with cell lines, animal models, and clinical trials concerning the effect of sulforaphane on regulation of gene activities through epigenetic modifications, including alterations of histone deacetylase activity and/or regulation of specific gene promoter methylation. It is interesting to attempt to clarify in what ways the SFN—competitive inhibitor of histone deacetylases—influences change of not only DNA methyltransferase expression but also expression of some tumour suppressor genes and genes specific for cell cycle regulation due to changes in methylation pattern of these gene promoters. From the literature data it seems that the sulforaphane delivered from cruciferous vegetables should be a very helpful natural compound whose consumption leads—via epigenetic modulation of gene transcriptional activity—to the reduction of cancer risk, or to a slowdown in cancer development as well as to intensification of efficacy of some standard chemotherapeutics.

## 2. Epigenetic Modifications Regulating Transcriptional Activity of Gene Promoters

### 2.1. Histone Deacetylation and the Role of Histone Deacetylase Inhibitors (HDIs)

Posttranslational covalent modification of nucleosome histone proteins, mainly acetylation and deacetylation, may play—apart from DNA methylation—an important role in chromatin structure formation. Acetylation of histones, catalyzed by histone acetyltransferases (HATs), causes change in the lysine's positive charge in histone proteins to neutral charge. The consequence of this charge change is inhibition of interaction between histone proteins and the negatively charged DNA chain, which causes nucleosome relaxation (“more open” chromatin) and increase in DNA accessibility for the transcriptional protein complex. The hyperacetylation of lysines on histone H3 and H4 is usually associated with actively transcribed genes [11]. During histone deacetylation, catalyzed by histone deacetylases (HDACs), acetyl groups are removed from lysine residues of core histones. That leads to chromatin condensation as a result of an increase in ionic interactions between positively charged lysine of histones with negatively charged DNA. The tightly packaged chromatin due to histone deacetylation is one of the well-established transcriptional inactivation mechanisms of tumour suppressor genes, such as *retinoblastoma*, *retinoic acid* β *receptor*, *p21*, *p53*, *p16*, *E-cadherin*, *RAR*β*2* and many others [11]. Many cancer malignancies are characterized by increased expression and activity of histone deacetylases (HDACs). The overactivity of HDACs, associated with transcriptional repression of tumour supressor genes, can lead to dysregulation of cell differentiation, cell cycle and apoptosis mechanisms, that finally can lead to cancer progression or to higher risk of cancer recurrence [12]. In studies with prostate cancer cell lines it has been documented that the activity of HDACs I, II and IV classes, those including Zn^2+^ ion in active pocket, may be competitively inhibited by synthetic compounds, such as trichostatin A (TSA), SAHA (suberoylanilide hydroxamic acid, also known as vorinostat), valproic acid or sodium butyrate [13]. Only sirtuins, which belong to class III of HDACs, *i.e.*, NAD^+^-dependent deacetylases, are not inhibited by the mentioned compounds [13]. Synthetic histone deacetylase inhibitors (HDIs) can cause an increase in a global protein acetylation as well as in histone acetylation. This inhibitory effect is dependent on time of exposure to HDIs, on the inhibitor concentration and on types of cancer cell lines. For example, HDIs have induced death of prostate cancer cells LnCaP and DU-145 but not PC3, whereas TSA has been more effective in inducing apoptosis, in LnCaP cells than in DU-145 cells [14]. Several results have shown that actions of HDIs are associated with reactivation of tumour suppressor genes and/or activity of genes encoding transcription factors. For example, in human gastric adenomas, carcinomas and human colon cancer, possessing reduced acetylated histone H3 and H4, 24h treatment with TSA led to an increase in global acetylation of histones and re-expression of *RAR*β*2* [15]. The retinoic acid receptor β2 negatively modulates expression of *DNMT1* by preventing formation of the AP-1 complex (Activator Protein 1 activates transcription of DNA methyltransferase) [16,17].

Another study has indicated that in colon cancer cell lines HT-29, human melanoma cell lines A375 and T24 bladder carcinoma cells, epigenetic suppression of *p21* is also directly linked to HDAC activity [18]. Although the epigenetic silencing of *p21* (possible to be reversed by HDIs) seems to be the main mechanism by which the *p21* gene is down-regulated in tumours, the gene promoter activity can be additionally regulated and controlled by various transcriptional activators, such as p53, Sp1/Sp3, E-boxes, STAT proteins, or repressors, e.g., c-Myc or FBI-1, having their response elements located in distal or proximal promoter region of *p21* [19,20,21,22,23].

Moreover, a deficiency or downregulation of *p21* correlates with tumour progression, aggressiveness and poor prognosis of various tumours, such as small-cell lung, colorectal, head and neck cancers [24]. It is important to emphasize that p21 tumour suppressor protein has a universal inhibitory activity towards CDKs (Cyclin Dependent Kinases)—critical proteins for cellular processes, such as cell cycle or transcription [19,25]. Studies with human fibroblasts have shown that the p21 is a component of a complex composed of p21, cyclin D1, PCNA (Proliferating Cell Nuclear Antigen), and CDKs. Formation of this complex plays a leading role in maintenance of the DNA methylation process [26].

However, some studies have shown that relatively few (approximately 10%) genes in cancer cell lines—such as leukemia, multiple myeloma and carcinomas of colon, bladder, kidney, prostate and breast, cultured for up to 48h with TSA, SAHA and other HDAC inhibitors—are directly altered in their expression [13].

Based on *in vitro* studies with animal models and on several clinical trials, it has been documented that tumour volume may be reduced by synthetic HDIs through cancer cell cycle arrest and induction of cell differentiation and apoptosis. SAHA is the first HDI approved by FDA for clinical use in cancer patients with cutaneous T-cell lymphoma. Although SAHA demonstrates activity against hematologic and solid cancers at doses well tolerated by patients, the results of clinical phase II trials have indicated that the response to orally-administered SAHA is only partial (24%) and approximately 45% of patients have not responded well (they have reported adverse skin effects such as pruritis) [13,27]. Synthetic HDIs are currently used as monotherapy or in combination with retinoids, taxols, gemcitabine, radiation *etc.*, in therapy of patients with hematologic and solid tumours, including cancer of lung, breast, pancreas, renal and bladder, melanoma, glioma, leukemia and lymphomas [3,13]. It is necessary to point out that, contrary to cancer cells, normal cells are relatively more resistant to SAHA and, despite increase in global acetylation, they do not respond to HDIs [13].

In spite of the fact that the inhibition of HDAC enzymes by synthetic compounds has been widely accepted as a cancer therapeutic strategy, many of these compounds exhibit also several associated side-effects and toxicities. For example, TSA is associated with developmental abnormalities, such as neural tube defects, whereas SAHA is related to several hematologic toxicities, such as myelosuppression and thrombocytopenia. Moreover, an oral administration of these synthetic HDIs is associated with anorexia, fatigue, and dehydration [27]. These side-effects and toxicities drive scientists to seek new HDIs in natural compounds delivered from commonly consumed vegetables. For this reason, the results with sulforaphane are considered promising.

### 2.2. Sulforaphane as HDI

Results of studies with colorectal and prostate cell lines indicate that chromatin architecture may be rearranged not only by synthetic histone deacetylase inhibitors (TSA, SAHA) but also by dietary nutrients delivered from cruciferous vegetables for example SFN and also its metabolites obtained from the mercapturic acid pathway in cells (such as SFN*-*cysteine, SFN-*N*-acetylcysteine) [2,10,28]. SFN and its metabolites, as competitive inhibitors, possess affinity for the active pocket of the HDAC enzymes through interaction with external amino acid residues located in the active site. TSA or SAHA displays a similar mechanism of interaction with HDAC enzymes [29,30]. The competitive inhibition of histone deacetylase activity by isothiocyanates influences transcriptional activity of genes, what has been indicated in *in vitro* and *in vivo* studies. [28]. It has been documented that treatment with SFN generates significant reduction of HDAC activity in lysate cells of HCT116 colon cancer, cancer cells of prostate, breast and human peripheral blood mononuclear cells [31].

Experiments with various prostate cancer cell lines, (BPH-1, LnCaP, and PC3) have indicated that at concentration of 15 μM SFN causes significant HDAC inhibition (by 30%–40%), which is accompanied by a 50%–100% increase in acetylation of histones. These changes are connected with G_2_/M arrest of cell development and induction of apoptosis in a caspase-dependent manner [32]. SFN also possesses the ability to change carcinogenic activity of xenobiotics by intensification of their metabolism through Nrf2-mediated induction of phase two detoxification enzymes followed by the induction of cell cycle arrest and apoptosis of various human cancer cell lines [10]. Inhibitory effect of SFN on HDAC activity has been noted also in studies with animal models that resulted in the slowing down of cancer development in a variety of organs, including breast, colon, lung, prostate and stomach. For example, treating mice with SFN for 21 days at a daily dose of 7.5 μmol per animal resulted in a 40% reduction of implanted prostate cancer PC-3 that is associated with both a decrease in HDAC activity and an increase in global histone acetylation [33]. Moreover, SKH-1 mice (hairless mice) treated with SFN have shown inhibition of chemically developed skin carcinogenesis, whereas extract of broccoli sprouts, containing high SFN level, has protected skin cells from the effects of UV radiation [34].

It is noteworthy that SFN effect includes also reactivation of *p21* transcription. The SFN effect on *p21* transcriptional reactivation is induced through hyperacetylation of histones H3 and H4 in the proximal *p21* promoter region containing the Sp1 binding site [35]. Recently it has been reported that MCF-7 and MDA-MB-231 breast cancer cell lines, exposed to 10 µM of SFN, demonstrate 2.5-fold and over 3-fold increase in *p21* mRNA levels, respectively [36]. Moreover, SFN (at the same concentration) used in combination with clofarabine (a new generation analogues of 2′-deoxyadenosine) improved the effect (2-fold) of the antileukemic drug on *p21* expression [36]. It is important to note that SFN had no effect on normal prostate epithelial cells [37] and negligible effects on normal cells of the breast cancer line, MCF10A as well [38].

MCF-7 cells treated with SFN demonstrated re-expression of the *RAR*β*2* gene [36]. Activation of *RAR*β*2* is associated with release of HDAC molecules from the nucleus and the initiation of PI3K/AKT signaling pathway [21].

To summarize, results of studies with synthetic and plant HDAC inhibitors suggest that SFN plays an important role in reactivation of genes (e.g., *p21* and *RAR*β*2*) silenced mainly due to deacetylation at the gene promoter regions [16,36,39]. The reactivation of *p21* and *RAR*β*2* genes by sulforaphane seems to be crucial actions in cells which, in consequence, should lead to reactivation of certain tumour suppressor genes. These suppressor genes are involved in a decrease in cell proliferation and/or in cancer progression.

### 2.3. DNA Methylation

Based on hitherto presented results it can be stated that synthetic HDIs play an important role in formation of relaxed (unpacked) chromatin structure, mainly due to posttranslational acetylation of histone proteins. It has been suggested several times that chromatin condensation can be an important factor effecting alteration of DNA methylation pattern. There has been evidence that there is close relationship between DNA methylation, histone deacetylation and condensed chromatin [40]. This strong interdependence has been noted for the first time in the context of inhibition of hypermethylated gene promoters. Initial opinions have indicated that DNA methylation and its interaction with MBP protein (binding methylated sequences CpG) is the primary event in mechanisms of silencing of gene activity and it is a signal for histone deacetylation, that finally results in chromatin condensation. The methyl binding protein contains two domains: methyl-CpG-binding domain and a transcriptional repression domain. Both of them can bind another protein called Sin3a, which interacts with histone deacetylase [41]. From here it can be suggested that DNA methylation is the dominant mechanism of transcriptional silencing system. However, subsequent research has shown that condensed chromatin structure that is a result of action of histone deacetylases can also be an important factor in establishment of DNA methylation patterns. The importance of this interdependence of epigenetic DNA modifications (methylation and deacetylation) and condensed chromatin structure is confirmed by results of research with colon cancer cells treated with HDAC inhibitors, where an increase in activity of genes silenced by epigenetic modification (for example, *MLH1*, *TIMP-3* and *CDKN2A*) has been possible only in the presence of DNA methyltransferase inhibitors [40]. Alterations in DNA methylation patterns, such as global DNA demethylation leading to chromosomal rearrangements and genome instability, as well as gene-specific promoter hypermethylation or hypomethylation contributing to gene silencing or activation, are potentially reversible and related to physiological, environmental and pharmacological factors, including diet. [36,42]. The establishment and maintenance of DNA methylation patterns are dependent on activity of DNA methyltransferases (DNMTs), catalyzing DNA methylation reaction. These enzymes have been reported to be overexpressed in many cancers, resulting in aberrant patterns of DNA methylation [43]. Since promoter hypermethylation may lead to silencing of tumour suppressor genes, DNA methylation and enzymes that catalyze this reaction have become important targets for epigenetic chemoprevention and anticancer therapy. The DNMT1 activity may be inhibited, for example, by synthetic 2′-deoxycytidine analogue (5-Aza-dC) which is strong competitive and irreversible inhibitor of the enzyme. 5-Aza-dC leads to direct inhibition of DNMT1 activity followed by inhibition of enzyme expression at mRNA and protein levels. The consequences of DNMT1 inhibition may be hypomethylation of promoters and reactivation of certain genes. It has been previously documented that in breast cancer MCF-7 cells treated with 5Aza-dC (0.6 µM, for 96 h) a significant decrease in promoter methylation of tumour suppressor genes, *i.e.*, *APC* (Adenomateus polyposis coli), *PTEN* (Phosphate and TENsin homologue) and *RAR*β*2* (retinoic acid receptor β2), is observed. Hypomethylations of these genes are connected with re-induction of their expression and also with 32% impairment of *DNMT1* expression [16,42]. Additionally, in tested breast cancer cells, 5-Aza-dC has caused an increase in *p21* expression (3.5-fold) [42]. On the other hand, in gastric cancer cells the *p21* gene promoter has been activated only by the HDIs but not by 5-Aza-dC [25]. Multiple studies with cancer cells exposed to DNMT inhibitors have shown an inverse correlation between *DNMT1* and *p21* expression [42,44]. However, in some cases the increase in *p21* expression has not been associated with alteration of the gene promoter methylation [23] and, for example, in the A549 cell line of human non-small cell lung cancer, an inhibition of DNMT resulted in rapid induction of *p21* expression by a DNA methylation-independent mechanism [45].

As reported a few years ago, and confirmed later by other authors, the treatment with HDIs causes DNMT1 to be competed out and released from the DNMT1/PCNA complex, followed by the binding of p21 to PCNA in the replication fork [26,46]. The removal of DNMT1 from the replication site can be a reason for global genomic hypomethylation and decrease in promoter methylation of certain genes that encode proteins important for cycle regulation and tumor suppression. It has been reported, for example, that some cancer cell lines (T24—bladder carcinoma cells, MDA-MB-231—breast cancer cells, Hep3B—liver cancer cells) demonstrate significant decrease in global methylation after TSA treatment. However, induction of acetylation and demethylation by TSA shows some gene selectivity and does not affect all methylated tumour suppressor genes equally [26,47]. In T24 and MDA-MB-231 cell lines treatment with TSA induces the expression at mRNA and protein levels of *E-cadherin* and *RAR*β*2* genes but the expression of *p16* is not induced [47]. Treatment of the cells with TSA alone also induced re-expression of *maspin* gene mRNA (mammary serine protease inhibitor) in MCF-7, T-47D, and ZR-75-1 breast cancer cell lines, but re-expression has not been observed in MDA-MB-231 and SK-BR-3 breast cancer cell lines. The combination of TSA and 5-Aza-dC in SK-BR-3 cell line activates *maspin* re-expression and enhances the gene re-expression in ZR-75-1 and BT-20 breast cancer cells caused by either agent alone [48]. It is important that the promoter demethylation of genes and their re-expression are shifted in time with respect to HDACs inhibition [16].

In addition to disturbance to DNMT1 binding to PCNA after the aforementioned HDIs treatment, alteration in DNMT1 activity may be a result of changes in protein mobility, distribution or expression level. In some authors’ opinion, an increase in local mobility of DNMT1 may result from reduction in chromatin compaction after TSA treatment or from hyperacetylation of DNMT1 [26].

The inhibition of HDAC is associated with decrease of nuclear DNMT1 protein level due to downregulation of *DNMT1* expression which has been observed in Jurkat leukemia T cells [49].

**Figure 1:**
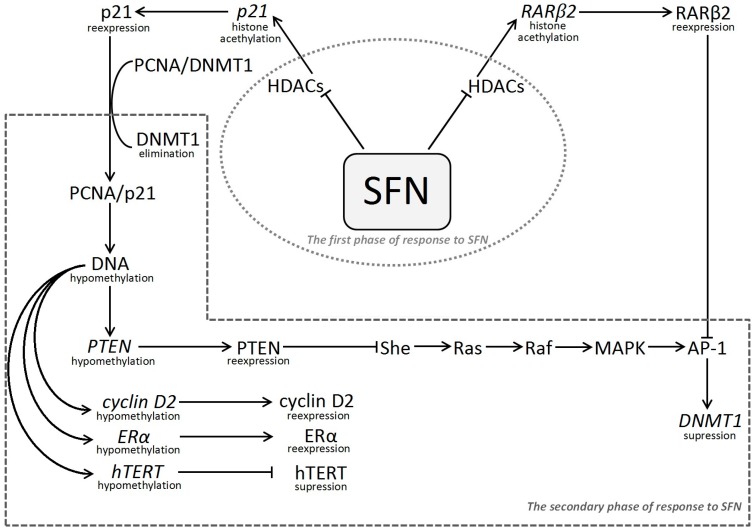
Schematic presentation of proposed molecular epigenetic mechanism of sulforaphane’s action including interdependence between histone modification and DNA methylation. SFN, sulforaphane; HDACs, histone deacetylases; RARβ2, nuclear retinoic acid receptor β2; AP-1, activator protein 1 (transcription factor); p21, cyclin-dependent kinase inhibitor 1; PCNA, proliferating cell nuclear antigen; DNMT1, DNA (cytosine-5-)-methyltransferase 1; PTEN, phosphate and TENsin homologue; MAPK Signaling Pathway (also known as the Ras-Raf-MEK-ERK pathway), the extracellular signal-regulated kinase pathway;—She, adaptor protein; Ras, GTPase (cellular signal transduction); Raf, kinase (activates MAP2K, which activates MAPK); MAPK, mitogen-activated protein kinase; Cyclin D2, member of the family of D-type cyclins; ERα, estrogen receptor alpha; hTERT human telomerase reverse transcriptase.

These observation lead to conclusion that effects of HDIs are not limited to direct histone deacetylation, but may indirectly change DNMT1 expression that can lead to hypomethylation and re-expression of the selected genes through pathways proposed in Figure 1.

### 2.4. Sulforaphane as Indirect Regulator of Promoter Methylation

Sulfpraphane’s inhibitory mechanism against HDACs activity is similar to TSA and SAHA, so SFN should also be involved both in a change of *DNMT1* (maintenance DNA methyltransferase 1) expression and in reactivation of methylation-silenced genes. Nevertheless, until now the sulforaphane influence on DNA methylation is not sufficiently clear. In one opinion, SFN can cause an increase in expression of genes with unmethylated promoters, but isothiocyanate is incapable to induce the re-expression of hypermethylated genes in cancer cells. However, in cancer cells treated with SFN, often a reduction of DNMT1 activity is observed, as documented in a study with human colon Caco-2 cells [50]. In LnCaP prostate cancer cells and in breast cancer cells SFN has caused a reduction of DNA methyltransferases expression, particularly *DNMT1* and *DNMT3a*. This has diminished the mRNA level of *DNMTs*, which are associated with re-expression of certain genes [44,51,52,53,54].

Previously it has been mentioned that SFN is involved in the detoxification process due to regulation of the expression of cellular defensive antioxidants and detoxification enzymes; in this process the Nrf2 transcription factor plays a crucial role. The treatment of prostate cancer cells from TRAMP mice (Transgenic Adenocarcinoma of the Mouse Prostate) and TRAMP-C1 cells with SFN has caused a decrease in the methylation ratio of the first five CpGs of the *Nrf2* gene promoter and the methylation has been reduced to 56% when SFN has been used at 1.0 and 2.5 μM concentration levels during five-day exposure. The gene promoter hypomethylation is associated with an increase in *Nrf2* mRNA expression and its downstream target *NQO1* on mRNA and protein levels. The SFN effect is also connected with a strong reduction of DNMTs expression at the protein level [54,55].

Other results have shown that SFN used at a dose of 30 μM for 48 h may down-regulate DNMTs and de-repress methylation-silenced *cyclin D2* expression [51]. The report is important because D2 protein belongs to D-type cyclin and is important in G1 to S phase transition. Dysregulation of these cyclins causes a disturbance in cell cycle control and promotes neoplastic transformation. Silencing of expression of *cyclin D2* through promoter hypermethylation is associated with cancer progression and aggressiveness in breast, lung, pancreatic and gastric cancers. These facts indicate that *cyclin D2* might act as a tumour suppressor gene in a cancer-type dependent manner. Authors of these studies suggest that the reactivation of the *cyclin D2* promoter results from SFN-mediated inhibition of both HDAC and DNMTs that leads to chromatin remodeling that facilitates access for various factors. The *cyclin D2* reactivation is associated with significant hypomethylation at the transcription factor c-Myc binding region which induces Sp1 transcriptional activity, although the selective SFN effect on demethylation of specific CpG sites is still unclear [51].

It has also been documented that treatment with SFN (10 μM) of MCF-7 cells (estrogen receptor ERα-positive cells) causes a decrease in methylation of tumour suppressor genes, such as *PTEN* by 25%, and *RAR*β*2* by 12% [36]. It is necessary to point out that these genes encode proteins indirectly implicated in *DNMT1* expression due to the following manner: PTEN reduces *DNMT1* expression by negative regulation of MAPK/AP-1 intracellular oncogenic signaling pathway, whereas *RAR*β*2* acts as suppressor factor for *DNMT1* transcription (Figure 1) [36,56]. Various effects of SFN (10 μM) on methylation of the mentioned genes have been accompanied with an increase in their expression: *PTEN* by 32%, and *RAR*β*2* by 55%–60%. Results for MDA-MB-231 cells (estrogen receptor ERα-negative cells) treated with SFN (10 μM), have indicated that reduction in *PTEN* promoter methylation is connected with the gene mRNA increase by 72%, and demethylation of *RAR*β*2* promoter by 25%, which is associated with a high increase in the gene mRNA expression (95%) [36]. It seems that the dramatic increase in *RAR*β*2* mRNA level, inadequate to demethylation degree of its promoter, can be attributed mainly to inhibition of HDAC activity followed by acetylation at the gene promoter region. The different effects of SFN on the expression of *PTEN* and *RAR*β*2* genes can also be dependent on the degree of *ERα* gene reactivation in these two breast cancer cell lines. The literature data indicate that SFN reactivates the *ERα* gene in both types of breast cancer cells (ERα-positive, and ERα-negative) [53].

Results of other studies have shown that SFN suppresses methylation in the *hTERT* (telomerase reverse transcriptase) promoter, which leads to transcriptional repression of the gene in breast cancer cell lines [38]. This fact is noteworthy, because a hypermethylatation of the regulatory region of *hTERT* is connected with increased expression of the gene, whereas hypomethylation of this regulatory region of *hTERT* decreases its transcription. This character of *hTERT* gene is in contrast to the common model of transcriptional gene activity regulation in which the methylation of cytosine in gene promoters results in gene silencing. The *hTERT* gene is frequently regulated in an epigenetic manner and is expressed in over 90% of human cancers, but not in normal somatic cells [38].

Some authors point out that decreases in *DNMTs* mRNA expression or protein may depend on the type of cancer cells, dose and time of exposure to the compound delivered from crucifers. For example, in LnCaP prostate cancer cells (androgen–dependent) SFN significantly decreases expression of *DNMT1* and *DNMT3b* on mRNA and protein level but the plant HDI has slight effect on DNMT1 protein level in PC3 and BPH-1 prostate cancer cells [51]. Simultaneously, the mentioned research indicates that SFN effect on DNMTs is different from the effect of 5-Aza-dC which inhibits (irreversibly, already after 24 h) activity of DNMT1. It has been also suggested that SFN effect cannot be associated with reduction of mRNA expression of the *DNMT1*. This fact has been confirmed by studies in which changes in methylation and expression of selected tumour suppressor genes in MCF-7 breast cancer cells exposed to SFN at 10 μM concentration for 96 h have not been associated with any relevant effects on *DNMT1* mRNA level [36].

Results of studies with mammalian cells treated with SFN indicate, that reduction of DNMT1 can run in indirect pathways and seems to be modulated by p21 after HDAC inhibition [44]. It is necessary to emphasize that treatment of breast cancer cell lines with HDIs and/or DNMT inhibitors lead to an increase in *p21* expression. It is possible that activation of the *p21* gene also depends on the methylation level of the STAT-binding site at the promoter, because hypermethylation of the *p21* gene at the proximal STAT-binding site correlates with decreases in *p21* expression [23]. Moreover, in the MCF-7 cells, the elevation of *p21* gene mRNA level is associated with no change of *DNMT1* mRNA level, in contrast to the effect of 5-Aza-dCyt [36,42].

In summary, most authors suggest that a downregulation of promoter methylation of certain genes, after treatment with SFN, results from decrease in *DNMT1* expression and from cross-talk between histone modification and DNA methylation. This cooperation can lead to new arrangement of chromatin structure, favorable towards factors enhancing the transcriptional process. It has been confirmed in studies showing that DNMT1 interact physically with HDAC1 or 2, and that DNMTs recruit class I HDACs to function as co-repressors in the transcription of tumour suppressor genes [57]. On the other hand, it is necessary to take into account the role of p21 protein, whose expression dramatically increases after treatment with SFN as well with other HDIs. Thus, it is most likely that after inhibition of HDACs activity there is a subsequent re-expression of *p21* at the mRNA and protein level. The reactivated p21 protein competes DNMT1 out from the replication complex [26,46]. This competition between DNMT1 and p21 for binding PCNA at the replicative fork results in reduction in promoter methylation of silencing genes. It is possible that there is also a decrease in *DNMT1* expression created probably either by reactivation of *RAR*β*2* due to HDAC inhibition, or by reactivation of *PTEN* blocking the intracellular signaling pathways MAPK/AP-1, or by other hitherto unknown mechanism. It seems that DNA hypomethylation is an effect subsequent to SFN action, where crucial roles are played by the DNMT1, p21, RARβ and PTEN proteins as illustrated in Figure 1.

## 3. Conclusions

The above-mentioned findings provide additional insight into the mechanisms by which SFN may act as a direct and/or indirect epigenetic modulator of gene transcriptional activity. The sulforaphane effect on regulation of gene promoter activity probably acts through a direct downregulation of its histone deacetylase activity followed by alternation in the gene promoter methylation in indirect ways.

Thus a consumption of cruciferous vegetables, treated as a source of bioactive isothiocyanate, such as sulforapane, seems to be potentially useful as an effective preventive factor reducing risk of cancer, or as a supply of nutrients slowing down cancer development or improving efficacy of standard chemotherapies. What should be noted, however, is that a critical determinant of SFN and other isothiocyanates efficacy is their bioavailability from dietary sources. A crucial factor in isothiocyanates absorption is release of isothiocyanates from their glucosinolate precursors by myrosinase. This process might be intensified by chopping or chewing of cruciferous vegetables, but on the other hand the enzyme can be inactivated by heat. In dietary supplements containing extracts of broccoli or other cruciferous vegetables the myrosinase is often inactivated and bioavailability of isothiocyanates from these supplements is limited. Results of studies devoted to evaluation of SFN absorption in healthy humans following consumption of broccoli sprouts and myrosinase-treated broccoli sprout extract indicate higher bioavailability of SFN from fresh broccoli sprouts. The determined levels of total SFN metabolites in plasma and urine have been significantly (3–5 times) higher in broccoli sprouts consumers compared to myrosinase-treated broccoli sprout extract consumers but neither of the two SFN forms has caused a significant decrease in HDAC activity. These observations indicate that the hydrolysis of glucosinolates to isothiocyanates is not the only factor influencing their absorption and, as authors suggest, food matrix and meal composition could also affect isothiocyanate absorption [58]. Further *in vivo* and *in vitro* studies are necessary to fully elucidate chemopreventive and anticancer properties of SFN and other isothiocyanates.
